# Multiple bilateral trigger fingers in a child with neurofibromatosis type I following an acute viral infection: A case report

**DOI:** 10.1016/j.ijscr.2020.04.020

**Published:** 2020-05-07

**Authors:** Mohammad M. Al-Qattan, Felwa A. AlMarshad, Attiya Ijaz, Qutaiba Shah Mardan

**Affiliations:** a King Saud University, Riyadh, Saudi Arabia; b King Faisal Specialist Hospital and Research Center, Riyadh, Saudi Arabia

**Keywords:** Trigger finger, Children, Neurofibromatosis, Viral infection

## Abstract

**Introduction:**

Triggering of the fingers in children is an uncommon entity. A review of the literature on the topic did not reveal any reports of multiple triggering in patients with neurofibromatosis.

**Case report:**

Our patient is a known case of neurofibromatosis type I (NF 1). At the age of 4 years, the child required chemotherapy to treat symptomatic bilateral optic glioma. Chemotherapy was complicated by severe viral upper respiratory tract infection with concurrent multi-joint synovitis and mild triggering of the fingers. A second course of chemotherapy was required 2 years later. This was also complicated by a viral infection, joint synovitis, and worsening of the triggering. Surgical release of the A1 and part of the A2 pulleys was curative.

**Discussion:**

Two factors contributed to the development of multiple triggering in our pediatric patient. The NF 1 itself predisposes to fibrosis. The second factor is the synovitis that accompanied the acute viral infection.

**Conclusion:**

We report on a case of multiple bilateral trigger fingers in a child with NF 1 following an acute viral infection and discuss the pathogenesis.

## Highlights

• We report on a case of multiple bilateral trigger fingers in a child with NF I.
• Triggering developed while the child was on chemotherapy following an acute viral infection.
• The pathogenesis is discussed.

## 1 Introduction

Triggering of the thumb is the most common presentation of digital triggering in children, and the condition may be self-limiting [1]. In contrast, triggering of the fingers in children is an uncommon entity and has been associated with multiple causes including storage diseases (such as mucopolysaccharidosis), anatomical abnormalities of the flexor digitorum superficialis and type I diabetes mellitus [2,3]. A review of the literature on the topic did not reveal any reports of multiple triggering of the digits in patients with neurofibromatosis. We report on a child with neurofibromatosis type I (NF 1) who developed multiple trigger fingers following an acute viral infection and discuss the possible pathogenesis. The work has been reported in line with the SCARE criteria [4].

## 2 Case report

**Fig. 1:**
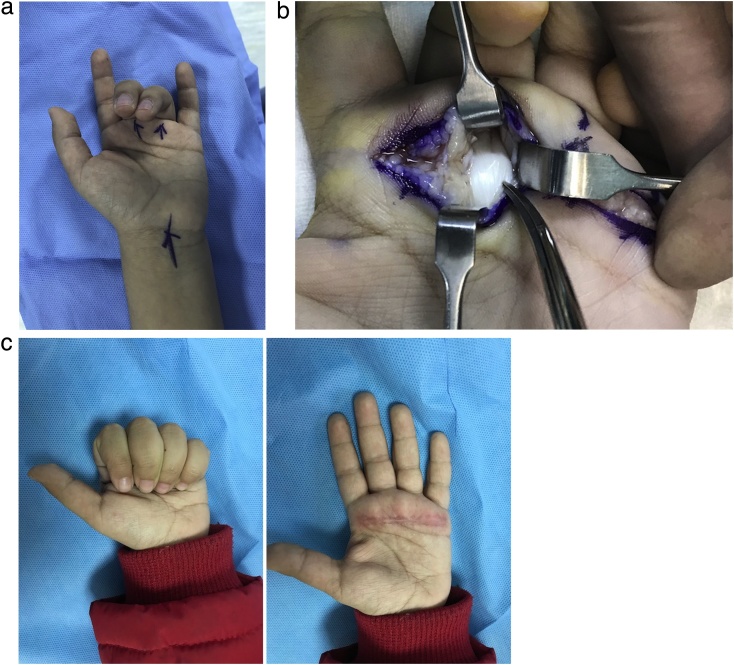
Demonstration of the clinical picture and the operative findings in the left hand. A) Preoperative appearance of the left hand. Note the locked Grade III triggering of the middle and ring fingers. There was also Grade II triggering of the index and little fingers. B) Following the release of pulleys, a large fibrous nodule was seen within the substance of the flexor tendon (at the tip of the mosquito). Note that the senior author uses a single transverse incision across the distal palm to obtain exposure to flexor tendons of adjacent fingers. C) Postoperative views showing full flexion and extension of the fingers without pain or locking.

Our patient was a Saudi girl with NF I. She had all of the classic features of NF 1 including multiple skin café au lait spots, multiple skin neurofibromas, Lisch nodules in the eye, and bilateral optic glioma. At the age of 4 years, the optic gliomas became symptomatic causing vision impairment. The patient was started on chemotherapy (Vincristine and Carboplatin). As a complication of the immunosuppression, the child developed a severe viral pharyngitis. The clinical diagnosis of viral rather than bacterial pharyngitis was done by the pediatrician and was supported by a negative bacterial throat cultures, the presence of nasal congestion, the presence of erythema without effusive or purulent exudate, and the absence of cervical lymphadenopathy. The viral infection was associated with multi-joint arthralgia and the development of triggering (Grades II- III as per grading system shown in Table 1) of all fingers of both hands. The child was given intravenous acyclovir (5 mg/Kg every 8 h for one week with recovery of the viral infection and the arthralgia. Triggering also improved and there was only mild residual triggering (Grade I-II) of the middle, ring fingers bilaterally. The optic gliomas were stable for the next two years, and there was no further change in visual acuity. At the age of 6 years, the gliomas started to grow in size again with further deterioration in vision. The patient was re-started on chemotherapy (Vincristine and Carboplatin), with the addition of Vinblastine. This was able to arrest the growth of the gliomas. However, the treatment was complicated with another severe viral pharyngitis. The degree of triggering became worse (Grades II-III); and all fingers (of both hands) became involved. Following the recovery of the viral infection, there was persistent triggering of the right index, middle, ring fingers (all were grade III), and the left index, middle, ring, little fingers (Grade II in the index and little fingers; and Grade III in the middle and ring fingers). The patient presented to the hand clinic at the age of 7 years and clinical examination confirmed that the triggering was at the A1 and the proximal part of the A2 pulley. The site of triggering was determined clinically by palpating the distal palm while the patient was moving the fingers: triggering was felt at both the distal palmar crease (site of the A1 pulley) and at the base of the proximal phalanx (the proximal part of the A2 pulley). Surgical release of the A1 and part of the A2 pulleys was done in two stages under general anesthesia (the right hand in the 1st operation and then the left hand in the 2nd operation). Operative findings were identical in all fingers: The pulleys were thick and there was a fibrous nodule within the substance of the flexor tendons in the area of the tight pulleys. Both the flexor superficialis and the profundus tendons had fibrous nodules. No other tendon abnormalities were seen such as proximal decussation or abnormal flexor superficialis slips. No debulking of the nodule was done, and the movement of all flexors was obtained with pulley release. No tissue was sent for histopathology. The patient did well after the surgery with complete disappearance of the triggering and complete recovery of range of motion of all fingers (Fig. 1).

**Table 1:** The grading of triggering used in the current report.

Grade of triggering	Description
Grade I	Pain and tenderness at the A1 pulley on moving the finger
Grade II	The digit “catches”, but the range of motion of the finger is complete without having to assist movement using the other hand
Grade III	The digit gets stuck (locked) in flexion. Extension is then possible by pushing the finger passively (using the other hand)
Grade V1	Fixed and locked finger (No range of motion) which is not passively correctable.

## 3 Discussion

We report on a unique case of triggering of the fingers in a child following chemotherapy and acute viral pharyngitis. We highlight the fact that there was partial spontaneous resolution of triggering after the first episode of viral pharyngitis, but the recurrent episode had a poor spontaneous recovery; necessitating surgical intervention. We also highlight the involvement of both flexor tendons at both the A1 and A2 pulleys; indicating the severity of the pathological fibrosis.

NF 1 is an autosomal dominant disorder caused by mutations of the *NF1* gene. Loss-of-function of the gene results in increased collagen deposition by fibroblasts [5]. This explains both the development of fibrous tumors and the predisposition to excessive fibrosis in patients with NF 1. The most common fibrous tumors in NF 1 are seen in the peripheral nerves (multiple neurofibromas), and plexiform neurofibromas have a risk of turning into neurofibrosarcoma. Intestinal fibromas [6], and interstitial pulmonary fibrosis (fibrosing alveolitis) [7,8] are other manifestation of excessive fibrosis in NF 1. Furthermore, abnormal skin wound healing with excessive collagen deposition has been demonstrated in *Nf1* knockout mice [9]. Given the fact that the pulleys of the flexor sheath are made of fibrous tissue, it is actually surprising that triggering is not commonly associated with NF 1.

The history in our patient indicates that the development of finger triggering is related to the acute viral illness which was complicated with what is known as transient synovitis of the joints and the flexor tendons (presenting as arthralgia and triggering). About 3% of children with acute viral infections develop transient synovitis of various joints, with the hip joint being the most commonly affected joint and result in the “limping child” [10,11]. Concurrent synovitis of the flexor tendons with digital triggering may also occur. Sharma et al. [12] reported on a child who developed multiple trigger fingers and joint synovitis following an acute viral upper respiratory tract infection. However, both the synovitis and the triggering are known to be transient and resolve spontaneously in these children. Triggering was relatively severe and did not resolve spontaneously in our case, perhaps because of the background of predisposition to excessive fibrosis associated with NF 1.

## 4 Conclusion

We report on a case of multiple bilateral trigger fingers in a child with NF 1 following two sessions of chemotherapy which were complicated by an acute viral infection. The clinical scenario was unique because there was partial spontaneous resolution of triggering after the first episode of viral pharyngitis, but the recurrent episode had a poor spontaneous recovery; necessitating surgical intervention. We also highlight the involvement of both flexor tendons at both the A1 and A2 pulleys; indicating the severity of the pathological fibrosis. Triggering in patients with NF 1 with viral infections while on chemotherapy has not been previously reported. We also discuss the pathogenesis of fibrosis in our case. The case has an important clinical implication regarding the need for hand examination in children with NF 1 on chemotherapy and who develop viral infections. This is specifically important in young children and other children with poor communication skills.

## Declaration of Competing Interest

None.

## Funding

None.

## Ethical approval

The study was approved by the research committee, National Hospital (Care), Riyadh, Saudi Arabia.

## Consent

Written informed consent was obtained from the parent for publication of this case report and accompanying images. A copy of the written consent is available for review by Editor-in-chief of this Journal on request.

## Author contribution

All authors contributed significantly and in agreement with the content of the manuscript. All authors participated in data collection and in writing of the manuscript. The senior author (MMA) performed the surgery.

## Registration of research studies

Not relevant here.

## Guarantor

M.M. Al-Qattan.

## Provenance and peer review

Not commissioned, externally peer-reviewed.

## References

[1] Mulpruek P., Prichasuk S. (1998). Spontaneous recovery of trigger thumbs in children. J. Hand Surg. Br..

[2] Cardon L.J., Ezaki M., Carter P.R. (1999). Trigger fingers in children. J. Hand Surg. Am..

[3] Yosipovitch G., Yosipovitch Z., Karp M., Mukamel M. (1990). Trigger finger in young patients with insulin dependent diabetes. J. Rheumatol..

[4] Agha R.A., Borrelli M.R., Farwana R., Koshy K., Fowler A., Orgill D.P., For the SCARE Group (2018). The SCARE 2018 statement: updating consensus surgical CAse REport (SCARE) guidelines. Int. J. Surg..

[5] Konomi H., Arima M., Tanaka H., Hayashi T., Ikeda S. (1989). Increased deposition of types III and V collagen in neurofibroma tissue from patients with von Recklinghausen disease. Brain Dev..

[6] Hashemian H. (1953). Familial fibromatosis of small intestine. Br. J. Surg..

[7] Massaro D., Katz S. (1966). Fibrosing alveolitis: its occurrence, roentgenographic, and pathologic features in von Recklinghausen’s neurofibromatosis. Am. Rev. Respir. Dis..

[8] Porterfield J.K., Pyeritz R.E., Traill T.A. (1986). Pulmonary hypertension and interstitial fibrosis in von Recklinghausen neurofibromatosis. Am. J. Med. Genet..

[9] Atit R.P., Crowe M.J., Greenhalgh D.G., Wenstrup R.J., Ratner N. (1999). The Nf1 tumor suppressor regulates mouse skin wound healing, fibroblast proliferation, and collagen deposited by fibroblasts. J. Invest. Dermatol..

[10] Landin L.A., Danielsson L.G., Wattsgård C. (1987). Transient synovitis of the hip. Its incidence, epidemiology and relation to Perthes’ disease. J. Bone Joint Surg. Br..

[11] Do T.T. (2000). Transient synovitis as a cause of painful limps in children. Curr. Opin. Pediatr..

[12] Sharma P.R., Gore S.M., Schreuder F.B. (2010). Bilateral trigger finger in a 7-year-old after a viral infection: case report. J. Hand Surg..

